# A longitudinal assessment of chronic care pathways in real-life: self-care and outcomes of chronic heart failure patients in Tuscany

**DOI:** 10.1186/s12913-022-08522-0

**Published:** 2022-09-10

**Authors:** E. Guidotti, F. Pennucci, A. Valleggi, S. De Rosis, C. Passino

**Affiliations:** 1 Management and Healthcare Laboratory, Institute of Management and Department EMbeDS, Scuola Superiore Sant’Anna, Scuola Superiore Sant’Anna, 56127 Pisa, Italy; 2 UOC Cardiologia e Medicina Cardiovascolare, Fondazione Toscana Gabriele Monasterio per la Ricerca Medica e di Sanità Pubblica, Pisa, Italy; 3 Institute of Life Sciences, Scuola Superiore Sant’Anna, Pisa, Italy

**Keywords:** Patient-reported outcomes measures (PROMs), Real-world evidence (RWE), Assessment, Monitoring, Adherence, Chronic care pathway, Chronic heart failure (CHF)

## Abstract

**Background:**

Worldwide healthcare systems face challenges in assessing and monitoring chronic care pathways and, even more, the value generated for patients. Patient-reported outcomes measures (PROMs) represent a valid Real-World Evidence (RWE) source to fully assess health systems’ performance in managing chronic care pathways.

**Methods:**

The originality of the study consists in the chance of adopting PROMs, as a longitudinal assessment tool for continuous monitoring of patients’ adherence to therapies and self-care behavior recommendations in clinical practice and as a chance to provide policy makers insights to improve chronic pathways adopting a patient perspective. The focus was on PROMs of patients with chronic heart failure (CHF) collected in the Gabriele Monasterio Tuscan Foundation (FTGM), a tertiary referral CHF centre in Pisa, Italy. During the hospital stay, CHF patients were enrolled and received a link (via SMS or email) to access to the first questionnaire. Follow-up questionnaires were sent 1, 7 and 12 months after the index hospitalisation. Professionals invited 200 patients to participate to PROMs surveys. 174 answers were digitally collected at baseline from 2018 to 2020 and analysed. Quantitative and qualitative analyses were conducted, using Chi2, t-tests and regression models together with narrative evidence from free text responses.

**Results:**

Both quantitative and qualitative results showed FTGM patients declared to strongly adhere to the pharmacological therapy across the entire pathway, while seemed less careful to adhere to self-care behavior recommendations (e.g., physical activity). CHF patients that performed adequate Self-Care Maintenance registered outcome improvements. Respondents declared to be supported by family members in managing their adherence.

**Conclusions:**

The features of such PROMs collection model are relevant for researchers, policymakers and for managers to implement interventions aimed at improving pathway adherence dimensions. Among those, behavioral economics interventions could be implemented to increase physical activity among CHF patients since proven successful in Tuscany. Strategies to increase territorial care and support patients’ caregivers in their daily support to patients’ adherence should be further explored. Systematic PROMs collection would allow to monitor changes in the whole pathway organization. This study brings opportunities for extending such monitoring systems to other organizations to allow for reliable benchmarking opportunities.

**Supplementary Information:**

The online version contains supplementary material available at 10.1186/s12913-022-08522-0.

## Background

Developed countries are facing several challenges in managing the raise of chronic diseases [1]. A major challenge is represented by worldwide system’s ability to assess chronic care pathways. Multiple ways for pathways’ assessment were employed (e.g., intervention rates, adverse events), however those are not capable of measuring all the pathways dimensions and even more important to capture those elements that really matters to patients over the whole care cycle [2]. Patient-reported outcomes measures (PROMs) represent a valid opportunity to reach fully assessment of chronic care pathways performance, by measuring health outcomes from the patients’ perspectives [3]. Furthermore, PROMs provide greater insight into the barriers that patients face in managing their health and a unique unbiased appraisal of the quality and efficacy of healthcare interventions [4]. Indeed, PROMs are an increasingly adopted Real-World Evidence (RWE) source to assess medical interventions and inform advances in quality healthcare [5], especially for chronic conditions as hearth failure [6]. RWE in medicine means evidence obtained from real world data (RWD), which are observational data during routine clinical practice [7] with a population-based approach [8]. RWD are routinely collected from a variety of sources, for example electronic health records, administrative data, product and disease registries, health surveys, patient-generated data including in home-use settings and data gathered from other sources that can inform on health status, such as mobile devices [9, 10]. In the last years, RWE has gained attention over the traditional ‘gold standard’ of evidence, namely Randomized Controlled Trials (RCTs) revealing that novel evidence sources are needed as complementary to RCTs. Among other, PROMs represent a fundamental RWE source for monitoring and evaluating healthcare practices, since capable of capturing otherwise unidentifiable care pathways dimensions (e.g., via administrative data or other RWE sources) [11]. However, despite the enormous potential, these surveys have been mainly used to measure short-term surgical outcomes or pathways, while little used for longitudinal outcome measurement and/or for chronic diseases monitoring, evaluation and management [12]. Chronic care pathways require longitudinal assessment of the care that patients receive, and of the outcome produced [13]. A key aspect in the chronic diseases’ management stands in the empowerment of patients, enhancement of their self-management and promotion of lifestyle changes. Some scholars suggest the need to shift from passive to more self-care based approaches focused on self-care maintenance, monitoring, and management [14–17]. Since chronic care diseases need a daily management that patients and families mainly carry out, collecting patients’ point of view over time is crucial, in order to give them a central role and responsibility in their care pathways [18]. Some examples of outcome measurements for long term and chronic patients are available, however mainly experimental, pilot or one-shot projects [13, 19, 20]. PROMs have the potential to systematically record patient health status during routine care, thus collecting information that could be useful for monitoring patients’ adherence to therapies and self-care behavior recommendations, that are fundamental to ensure good outcomes [19]. The World Health Organization defined adherence to long-term therapy as: ‘the extent to which a person’s behaviour – taking medication, following a diet, and/or executing lifestyle changes, corresponds with agreed recommendations from a health care provider’. Indeed, adherence does not refer only to medication, it encompasses numerous health-related behaviours that extend beyond taking prescribed pharmaceuticals (e.g., attending follow-up appointments, and executing behavioural modifications that address personal hygiene, smoking, unhealthy diet and insufficient levels of physical activity are all examples of therapeutic behaviours, hereby called lifestyle indications). The importance of treatment adherence can be summarized as: better adherence would promote better outcomes. On the contrary, poor adherence leads to worsening of the disease, increased mortality and substantial rise in health care costs [20]. There is not a unique practice for measuring adherence behavior and several strategies were adopted across time. Among others, a bunch of literature reported of PROMs adoption for measuring medication adherence, however few focused on chronic care pathologies other than cancer and the assessment emerged as not continuous or systematic [20, 21].

This study aims at exploring wether chronic heart failure (CHF) patients’ adherence to pharmaceutical therapies and self-care behavior recommendations changes across their care pathway and time, by also inquiring the effects obtained throught the use of healthcare services involved in the pathway. RWE from different sources was used to fully monitor and evaluate the chronic care pathway. The specific focus will be on PROMs with further information drawn from the patients’ reported experience measures (PREMs) across their care pathway. PREMs are tools that allow to gather information on patients’ views of their experience while receiving care [22]. Suggestions to policy makers and healthcare managers are provided to further improve the chronic heart failure pathway from a patient perspective.

## Methods

### Setting - FTGM, a center of excellence

The study was conducted in the Gabriele Monasterio Tuscan Foundation (FTGM), a tertiary referral centre for CHF based in Pisa, Italy. FTGM ranks first in Italy for cardiological interventional procedures, second for the complexity of cases treated and fifth for paediatric surgery. Furthermore, the hospital records a remarkable performance in the adherence to pharmacological therapies as visible on the Interregional Performance Evaluation System (IRPES) headed by the Management and Healthcare Laboratory (MeS Lab) of the Sant’Anna School of Advanced Studies in Pisa [23, 24]. In FTGM, the chronic heart failure PROMs and PREMs programme was developed, first, as a pilot study and part of a wider ongoing collection of PROMs and PREMs for other specialties (e.g., elective orthopaedic surgery, breast cancer surgery) in several hospitals across Tuscany region and other Italian institutions [18, 25–28]. Then, PROMs became a part of the routine way of providing care. All PROMs and PREMs programmes are coordinated by the Management and Healthcare Laboratory (MeS Lab) of the Sant ‘Anna School of Advanced Studies in Pisa [29] [30] Survey administration, questionnaires characteristics and data reporting.

Data collection started in 2018 with no interruption and is conducted digitally. CHF Patients are enrolled and receive the first questionnaire after an acute event with hospitalization in FTGM. Follow-up questionnaires are sent 1, 7 and 12 months after index hospitalisation. A detailed description of the patients’ enrolment and survey administration is available in Pennucci and colleagues [18].

Questionnaires include measures of outcomes, experience of care and patient adherence to pharmaceutical and lifestyle guidelines. As for PROMs, the Italian short version of the Kansas City Cardiomyopathy Questionnaire-12 (KCCQ-12) is used to measure disease-specific outcomes [31]. The KCCQ-12 scale measures several CHF dimensions: physical limitation, symptoms frequency, social limitation, and quality of life. The total score goes from 0 (worst possible condition) to 100 (best possible condition). The KCCQ-12 questionnaire is administered at each follow-up time point, however not in the baseline questionnaire as recommended by the International Consortium for Health Outcomes Measurement guidelines [18, 32]. In order to detect changes in outcome, each follow up score is compared to the first score reported at 1 month time point after discharge. The difference between scores is used in the analyses as a specific variable that ranges from negative to positive values since there could be a worsening in outcome.

The Italian version of the Self-Care Heart Failure Index (SCHFI) is administered at each time point (see Table S1 in Additional file 1). Such questionnaire assesses both adherence to medicines and self-care behavior recommendations and is organized in three areas: Self-Care Maintenance (ten items considering all those behaviours that monitor signs and symptoms and maintain HF stable), Self-Care Management (in the subset of patients with dyspnoea or oedema during the previous month: six items measuring symptoms recognition and responses to signs and symptoms of an exacerbation), and Self-Care Confidence (six items assessing patient self-efficacy in performing the entire self-care process). Further details on the SCHFI are available here [18, 33]. PREMs questionnaire is submitted at each time point as well. At baseline, patients are asked about the quality of care before the index hospitalisation and during the hospital stay. The questionnaire is structured into nine items, three of them concerns the pharmaceutical dimension. All the items were described elsewhere [18]. One month after the hospitalization, the questions mainly concern the hospitalization experience, management of discharge and home care organization. At 7 and 12 months after baseline hospitalization, questionnaire explores clinicians’ monitoring, follow-up care coordination and visits, home care management, out-of-pocket expenditure, acute events occurrence and four items concern the pharmaceutical dimension. For the analyses presented in this paper, a selection of questions was made considering the impact of the integration of professionals, clarity of information and drug therapy management on adherence and outcomes [34] (see Table S2 in Additional file 1).

**Fig. 1:**
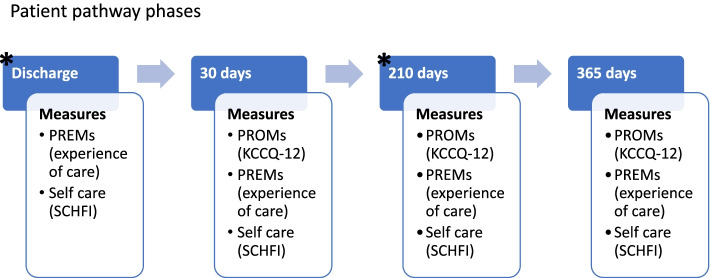
represents the questionnaires’ timeline with a synthetic list of measures. Stars indicate the routine visits scheduled in a usual pathway of FTGM patient with chronic failure

Figure 1 represents the timeline for administering the questionnaires with a synthetic list of measures as further explained by Pennucci et al. [18]. It is worth mentioning that FTGM doctors and nurses have a direct interaction with the patient that includes counselling moments (e.g., professionals dedicate time to patients to explain the importance of drug therapies and lifestyle guidelines adherence) during the hospitalization and 6 months after discharge when a check appointment is scheduled as highlighted through the asterisks in the Figure below.

The first questionnaire includes questions on socio-demographic characteristics of patients. Comorbidities of patients are assessed by clinicians, who report them during the enrolment phase by selecting the specific diseases resulting from his/her anamnesis in a list.

### Analysis of responses

#### Quantitative analysis

In order to measure patients’ adherence in terms of pharmaceutical and self-care behavior recommendations, both SCHFI scale and some additional questions on drug therapy composition and management were used.

More in details, the additional questions are shown in Additional file 2.

Descriptive statistics and multivariate analyses were conducted considering single variables on socio-demographic characteristics, self-care behavior, use of and interaction with healthcare services. Chi2 and t-tests were used to detect statistically significant differences. All the analyses were conducted both on the composite score and the single items of SCHFI scales (see Table S1 in Additional file 1).

Nine multivariate linear regression models were estimated, incrementally including the independent variables. Three independent variables were considered: Self-Care Confidence change at 12 months; Self-Care Maintenance change at 12 months; and KCCQ-12 change at 7 months. For the outcome, we selected the 7 month follow up based on the precedent analyses. For each independent variable, three models were estimated considering: the impact of the other self-care or outcome variables; socio-demographic and lifestyle characteristics of patients (age, gender, education, physical activity, smoking); all the baseline conditions of patients reported by clinicians (NYHA class, time since diagnosis, comorbidities, and drug therapy).

All the analyses have been conducted using the software STATA15.

#### Qualitative analysis: anecdotal evidence

Three open-ended questions populate the survey at different time points. In those sections, respondents are requested to tell their experience. More specifically, those sections are dedicated to the ‘storytelling’. Open-ended questions are intended to gather several insights on respondents’ personal experiences across the patient journey: hospitalization experience, post-discharge experience, home care management, pharmaceutical and lifestyle habits adherence, family support. Furthermore, it is a chance for patients to highlight who better support them in their care path. Open-ended questions’ comments were processed using NVivo software (NVivo10®), after being manually analysed by two researchers. A specific node was created for each open-ended question at different time points, thus resulting in six final nodes. Sub-nodes were generated for specific topics of interest (e.g., male versus female answers). The final coding was reviewed, and the node network was checked by a researcher; discussion between investigators was conducted for any misalignment. As part of the analysis, word frequency analysis was conducted though the software at each node.

## Results

The result section is organised following the patients’ care pathway through the monitoring of PROMs questionnaires that include both quantitative and qualitative information. The quantitative results are firstly presented: the first subsection will be dedicated to describing patients’ characteristics and baseline condition, together with reported medicines and self-care behavior recommendations adherence; the subsections dedicated to follow up questionnaires will be oriented to report also differences from baseline and correlations among self-care and outcome. Afterward, qualitative results are reported with a similar scheme.

Professionals invited 200 patients to participate into the PROMs surveys. Table 1 reports the number of patients who replied over time. 174 valid answers were collected at baseline from 2018 to 2020 and analysed in this paper.

**Table 1:** Reports respondents to FTGM CHF PROMs’ over time

	**Enrollment**	**Discharge (T0)**	**30 days (T1)**	**7 months (T2)**	**12 months (T3)**
**Number of respondents**	200	174	151	130	122

### Baseline questionnaire information

Most of the 174 respondents filled in the first questionnaire on their own (60%). Most of them were males, aged over 70 and retired. In terms of family and social support, most of the patients were not living alone (87%) and overall, 91.95% of patients could count on partner or relatives’ support. Around 50.00% of patients reported to provide support to others as well.

On average, patients have had heart failure for 8 years and more than half of them (52%) answered the questionnaire because of a re-hospitalization due to heart failure de-compensation. 66% of patients were in NYHA class II. Both patients and professionals reported, on average, the co-existence of other 2 diseases together with heart failure.

In terms of self-care behavior recommendations, 60% of patients were used to smoke while 36.00% reported to have never smoked before. The majority of patients did not regularly perform any physical activity during their weekly routine (46.60%), with males being more active than females (*p* = 0.02). The average BMI was slightly higher than the established normal threshold and there was not a high deviation on this measure. Further details on socio-demographics are reported in Table 2.

**Table 2:** Reports FTGM CHF PROMs’ sample size demographics at baseline

	**RESPONSES** ***N*** **= 174**
**Age (average and %)**	71 ± 11 (min 29 - max 93)
**Under 55**	8.00
**55–64**	20.50
**65–79**	46.50
**Over 80**	25.00
**Gender (%)**	
**M**	75.86
**F**	24.14
**BMI (average)**	26.4 ± 4.90 (min 15.40 - max 45.20)
**Education (%)**	
**No title/Primary school**	22.41
**Secondary school diploma**	31.03
**High school diploma**	35.06
**Degree or more**	11.49
**Occupational Status (%)**	
**Housewife**	4.02
**Not occupied**	1.72
**Atypical contract**	0.57
**Permanent contract**	13.79
**Freelancer**	8.62
**Artisan or similar**	2.30
**Retired**	64.94
**Other**	4.02
**Smoking habit (%)**	
**Never smoked**	35.63
**Ex-smoker**	58.05
**> 20 cigarettes a day**	6.32
**Physical activity per week (%)**	
**No physical activity**	46.55
**30 minutes**	22.41
**1 hour**	11.49
**2 hours**	8.62
** > 2 hours**	10.92

In terms of drug therapy, at baseline professionals reported that patients were assuming on average 4 different drugs daily. Patients reported the same information in their questionnaire, with 90. % of them never forgetting to take their medication. Of the 16 patients who declare to forget to take their medication, only 3 confirmed to forget it more than once a week. The average SCHFI score on Self-Care Maintenance is 53.04, while the average score on Self-care Confidence is 62.59 over a range that goes from 0 to 100. Older patients use memos to remember their medication more often than younger patients (*r* = 0.19, *p* < 0.02), while they are a bit less confident in their ability to manage heart failure symptoms (*r* = − 0.14, *p* < 0.08). Patients who live alone tend to be less adherent in avoiding to getting ill (*p* < 0.03).

Answering to this first questionnaire, patients declared that generally they are followed by a cardiologist working inside the hospital of admission (46.70%) and sometimes by the General Practitioner (GP) (21.60%). More than the half of patients reported that GPs are not in contact with specialists in following their patients (62.00%).

### First follow up questionnaire: one month after the hospitalisation

Drug treatment for heart failure was not changed for the 78% of respondents, while another 20% had a change from the specialist. Considering self-care behavior recommendations adherence, 90% of patients reported to have attended follow up visits. 80% of patients declared that their GP was aware of the hospitalisation, but only 42% of them had a contact with him during this period.

The average SCHFI score on Self-Care Maintenance is 61.58 (+ 7.39 on average compared to baseline), while the average score on Self-care Confidence is 61.71 (− 1.70 on average compared to baseline) over a range that goes from 0 to 100. Analysing the cohort of patients who answered to all the three follow up questionnaires, it emerges that 62.50% of them had an improved score on the Self-Care Maintenance scale and 45.88% on the Confidence scale comparing baseline and 30-day follow up. These two measures are weakly correlated according to Pearson’s r (0.23, *p* < 0.01).

The Maintenance items with a higher score on the alternative “Always” are “Attending the follow up” and “Take my medication” (59.80 and 91.20% respectively). On the Confidence dimension, “Following the received advice” and “Detecting changes in my health status” show a higher selection of “Nearly always” alternative (81 and 44% respectively).

Association between baseline characteristics of patients and self-care behavior recommendations adherence of patients was controlled. Age of patients is positively associated with avoiding to getting ill (*r* = 0.17, *p* < 0.05) and with a more frequent use of memos to take medication (*r* = 0.18, *p* < 0.05). At the same time, older patients report a lower level of physical activity (*r* = − 0.19, *p* < 0.03). Males report a slightly lower confidence in evaluating the severity of symptoms (*p* = 0.08). No statistically significant association has been found considering patients’ characteristics and the overall score on Maintenance and Confidence scales.

30 days after baseline questionnaire patients report also on their health outcome: the average baseline KCCQ-12 score is 64.49 on a maximum of 100. A weak association was found between Self-Care Maintenance score and KCCQ-12 score (*r* = 0.15, *p* = 0.05). However, an improvement on the overall Maintenance score is not associated with an improvement in KCCQ-12 score.

### Second follow up questionnaire: seven months after the hospitalisation

After seven months from the hospitalisation, more patients were followed by the GP (28.60%) and also by a cardiologist working inside the hospital (48.45%). The proportion of patients selecting the option “Never” regarding the frequency of interaction between GP and specialist in following their case dropped from 62 to 21% considering the baseline reporting of patients.

Regarding the drug therapy, a higher proportion of patients reported to take from 5 to 7 different drugs a day compared to the baseline questionnaire (+ 10%). 40% of them declared to have had a switch of therapy from their specialist, but the majority had a continuity in drug therapy (56.5%). Only 12% of patients report to have used home assistance services. Patients continue to attend follow up visits in the 92% of cases.

At this follow up, the average SCHFI score on Self-Care Maintenance is 63.11 (+ 7.90 on average compared to baseline), while the average score on Self-care Confidence is 64.16 (− 0.18 on average compared to baseline). A slightly higher proportion of patients reported an improvement on the Maintenance score (65.85%) and on the Confidence score (50.62%). A stronger correlation emerges between the two scores 7 months after the admission, with a 0.37 value for Pearson’s r (*p* < 0.001).

Considering the single items of both the Maintenance and the Confidence scales, the results on the more frequently adopted behaviours are confirmed with respect to the 30-days follow up. More details are shown in Additional file 3. No statistically significant association has been found at this follow up with age, while it emerges that males are more used to exercise 30 minutes a day (*p* = 0.05) and to use less frequently a memo to take medication (*p* = 0.02). The same pattern emerges for patients who live alone, that use less memo for taking medication (*p* < 0.02).

**Fig. 2:**
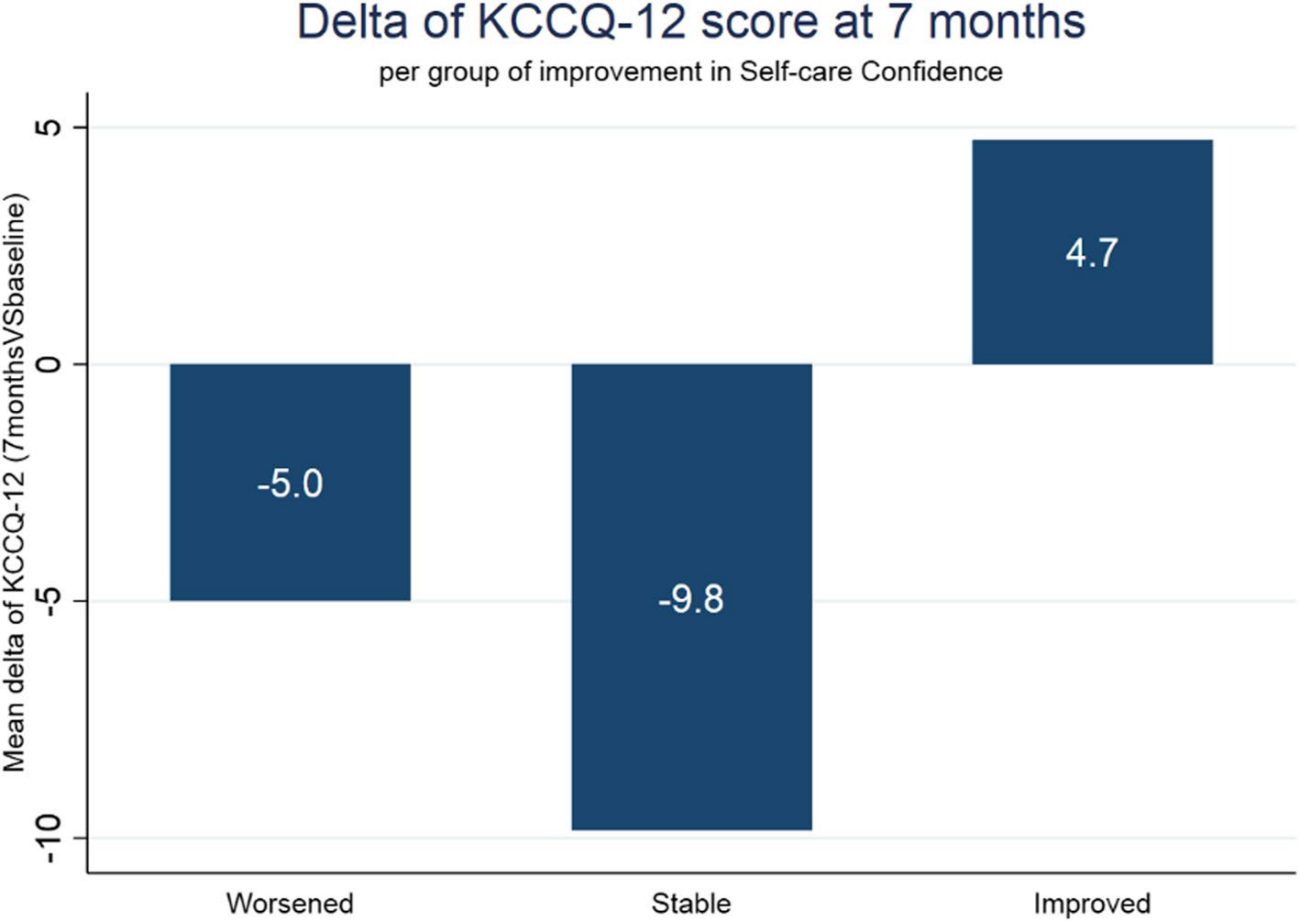
Represents the average difference in KCCQ-12 outcome score considering three groups of patients: those who registered worsening, stability, and improvement in the self-care Confidence

The questionnaire filled in 7 months after the hospitalisation includes the first follow up measure of KCCQ-12 score, that is on average 63.94 (− 0.54 on average compared to baseline). No association was found between this score and overall score on Self-Care Maintenance and Confidence. However, a statistically significant difference was found at the 7 months follow up comparing patients who had an improvement in the self-care Confidence dimension and the other patients (Fig. 2): who improves the confidence in self-caring tends to have a better results 7 months after the discharge from the hospital (*p* = 0.007).

**Fig. 3:**
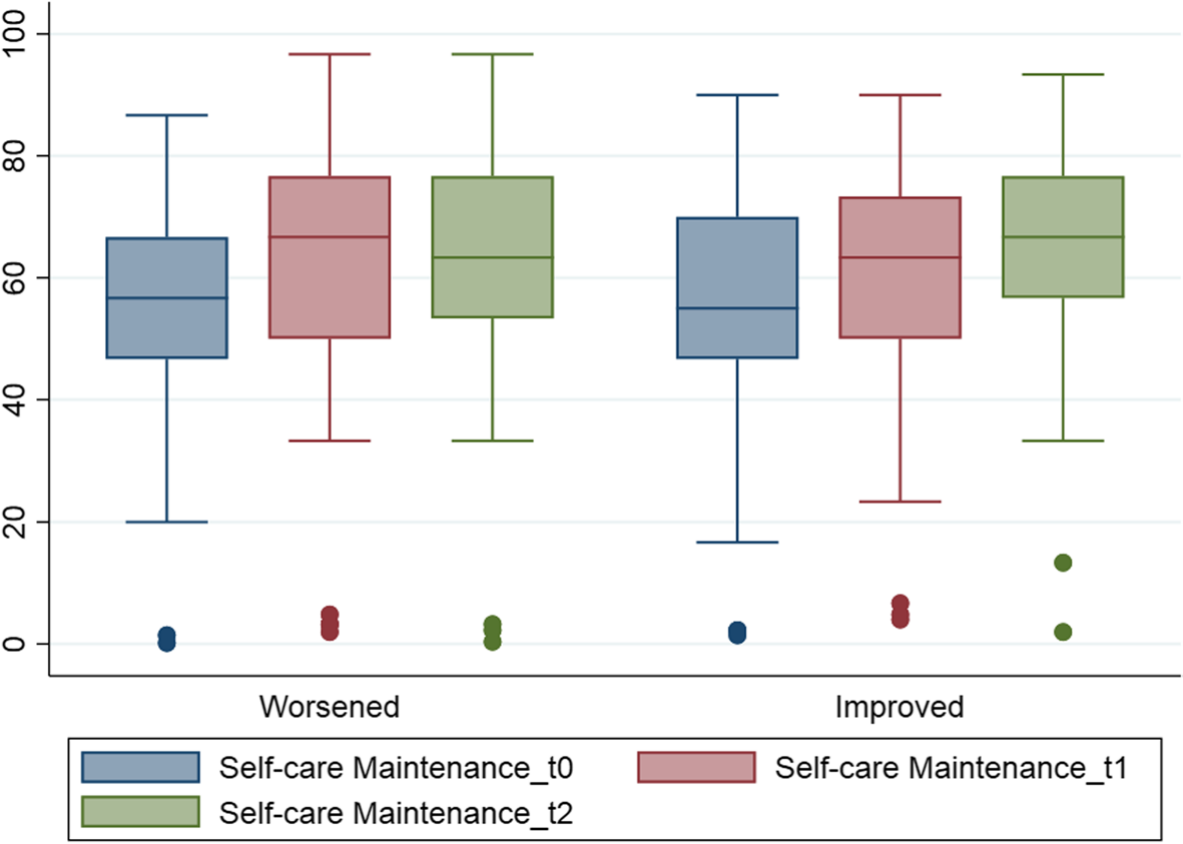
Box plot of Self-care Maintenance score distribution over group of patients who had a worsened or improved KCCQ-12 score 7 months after the hospitalisation

Figure 3 represents the average score on Self-Care Maintenance from baseline to seven-months considering two group of patients: first, who reported a worsening on their KCCQ-12 score; and second, who reported an improvement. Who experienced an increase in the Self-Care Maintenance, is more likely to have stable or improved health outcomes.

### Third follow up questionnaire: twelve months after the hospitalisation

One year after the baseline hospitalisation, the GP followed a slightly higher percentage of patients (30.40%), while the cardiologist working into the hospital followed a slightly lower percentage of patients (46.20%). The proportion of patients selecting the option “Never” in describing how often GPs and specialists interacted is again lower than the previous follow-up (17.00%).

One out of four patients had to take 8 or 9 different drugs a day, but around 70% of them continued to follow the same drug therapy. Pharmaceutical adherence was reported to be very high, with 93% of patients never forgetting to take medication. On the other side, a lower proportion of patients declared to regularly attend follow up exams (84%) and to have received home assistance services (10.40%).

SCHFI score on Self-Care Maintenance is on average 58.61 (+ 2 on average compared to baseline) and SCHFI score on Self-care Confidence is 66.06 (+ 1.35 on average compared to baseline). More than the half of patients reported an improved score both on Maintenance (61.22%) and on Confidence (51%). No correlation was found between the two scores at this follow up.

Younger patients were more adherent in checking if their ankles were swollen (*p* = 0.05) and in doing physical activity (*p* = 0.03). It was confirmed that males tend to use less frequently a memo to take medication (*p* = 0.02).

Patient-reported average outcome score, measured with KCCQ 1 year after the hospitalisation, is 66.22 (− 0.72 on average compared to baseline). No association was found between this score and overall score on self-care Maintenance and Confidence.

### Putting all together: self-care and outcome over time

Table 3 reports the average score of SCHFI scales on self-care Maintenance and Confidence over time. As evident, the SCHFI Maintenance score tends to improve from the baseline to 7 months, while the Confidence one tends to be higher 1 year after the first reply of patients. Most of the patients tend to improve over time on the Maintenance SCHFI score (more than 60% of patients improve at each follow up), while for the Confidence SCHFI score there was more polarization (from 45 to 51% of patients improved at each follow up).

**Table 3:** Reports the average score of SCHFI scales on Self-Care Maintenance and Confidence over time

	**Discharge (T0)**	**30 days (T1)**	**7 months (T2)**	**12 months (T3)**
**SCHFI Maintenance (average score)**	53.04 ± 20.27 (min .16 - max 96.66)	61.58 ± 21.47 (min 1.96 - max 99.99)	63.11 ± 20.53 (min .36 - max 96.66)	58.61 ± 26.07 (min .64 - max 99.99)
**SCHFI Confidence (average score)**	62.59 ± 23.38 (min .01 - max 100)	61.71 ± 24.76 (min .09 - max 100)	64.16 ± 24.99 (min .09 - max 100)	66.06 ± 26.35 (min .36 – max 100)

Filling in the KCCQ-12 questionnaires, patients reported their outcomes over time. Table 4 reports the average scores.

**Table 4:** Reports the average KCCQ-12 overall and sub-dimensions scores

	**30 days (T1)**	**7 months (T2)**	**12 months (T3)**
**KCCQ-12 TOTAL (average)**	64.49 ± 24.71 (min 6.25 - max 100)	63.94 ± 26.60 (min 6.77 - max 100)	66.22 ± 26.57 (min 0 - max 100)
**KCCQ-12 Physical limitation**	62.46 ± 30.33 (min 0 - max 100)	60.03 ± 32.74 (min 0 - max 100)	62.89 ± 31.02 (min 0 - max 100)
**KCCQ-12 Symptoms frequency**	78.60 ± 21.88 (min 0 - max 100)	77.28 ± 23.07 (min 8 - max 100)	77.51 ± 25.09 (min 0 - max 100)
**KCCQ-12 Quality of life**	57.79 ± 28.90 (min 0 - max 100)	60.77 ± 30.97 (min 0 - max 100)	63.05 ± 30.61 (min 0 - max 100)
**KCCQ-12 Social limitation**	58.72 ± 31.08 (min 0 - max 100)	57.75 ± 32.19 (min 0 - max 100)	60.18 ± 32.65 (min 0 - max 100)

N.B. Both 7 and 12 months scores are reported for who replied both to this and the first questionnaire

### Multivariate models

Table 5 illustrates the results for the nine estimated multivariate regression models.

**Table 5:** Results of multivariate regression models. Grey cells indicate the variables not considered in each model. SC=Self Care

Dependent variable	Independent variables	**Model 1**		**Model 2**		**Model 3**	
		Coeff	*p*Val	Coeff	*p*Val	Coeff	*p*Val
SC Confidence change at 12 months	SC Maintenance change at 1 month	−.37	**0.008**	−.34	**0.017**	−.37	**0.014**
	SC Maintenance change at 6 month	.42	**0.017**	.43	**0.016**	.40	**0.029**
	SC Maintenance change at 12 month	−.58	0.644	−.95	0.469	−.05	0.678
	Female			6.71	0.411		
	Age			.27	0.395		
	Education			−4.55	0.263		
	Physical activity			3.48	0.212		
	Smoking			4.18	0.494		
	NYHA class					−.58	0.993
	Time since diagnosis					.04	0.937
	Average n. comorbidities					−.42	0.894
	Average n. drugs					−1.88	0.530
SC Maintenance change at 12 months	SC Confidence change at 1 month	−.32	0.817	−.08	0.527	−.018	0.901
	SC Confidence change at 6 month	.11	0.442	.10	0.485	.098	0.525
	SC Confidence change at 12 month	−.04	0.739	−.5	0.702	−.04	0.765
	Female			.02	0.958		
	Age			−.14	0.987		
	Education			−8.35	**0.051**		
	Physical activity			−3.83	0.200		
	Smoking			−1.41	0.828		
	NYHA class					12.9	**0.057**
	Time since diagnosis					.18	0.738
	Average n. comorbidities					−.058	0.986
	Average n. drugs					−.19	0.952
KCCQ-12 change at 7 months	SC Confidence change at 1 month	−.025	0.720				
	SC Confidence change at 6 month	.18	**0.010**	.20	**0.001**	.18	**0.004**
	SC Maintenance change at 1 month	−.11	0.177				
	SC Maintenance change at 6 month	.024	0.781	.001	0.983	−.022	0.756
	Female			3.44	0.396		
	Age			−2.12	0.284		
	Education			−2.12	0.284		
	Physical activity			3.10	**0.024**		
	Smoking			1.48	0.624		
	NYHA class					.87	0.777
	Time since diagnosis					.008	0.975
	Average n. comorbidities					−2.56	**0.059**
	Average n. drugs					1.42	0.349

A first set of models were estimated considering Self-Care Maintenance and Confidence change over time as independent variables. These two variables regressed significantly one on the other. In particular, 7 months after the hospitalisation they had a direct relationship, while 12 months after it emerges an indirect pattern. Maintenance augments if the NHYA class of the patient is higher (coeff. 12.90, *p* = 0.05).

Considering the change of KCCQ-12 7 months after the hospitalisation as dependent variable, the only self-care independent variable impacting is the change in self-care Confidence 7 months after the hospitalisation (coeff .18, *p* = 0.004). This also holds true if socio-demographic characteristics of patients are included into the model. A further significant impact comes from the physical activity level reported at baseline by patients: higher level of physical activity resulted in higher improvement in outcome (coeff. 3.10, *p* = 0.02). As expected, higher number of comorbidities resulted in low improvement in the outcome score 7 months after the hospitalisation (coeff. -2.56, *p* = 0.05). None of the variables describing the interaction of patients with health care services result as significant regressor of change in outcome 7 months after the hospitalisation.

### Anecdotal evidence from open comments

Anecdotal evidence from open comments analysis of comments received at FTGM shows a large group of patients or carers decided to provide narrative comments, mostly positive ones. The analysis of comments carried out via a text analysis software highlighted the most common terms in storytelling emerged to be professionalism, availability, kindness, and expertise. Specifically, the focus of the comments changed according to the type of question and the time of administration.

As regard the hospital stay, typical comments 30 days after discharge concerned patients’ experience during the hospitalization, with a majority of positive comments. Respondents expressed gratitude for the expertise, availability, kindness, and humanity of professionals, both doctors and nurses (Table 1). Here below, few examples of positive patients’ comments:

‘Doctors and nurses have always been professional and helpful. I always felt like I was in the best facility to face my health issue’.

‘Doctors were very prepared, excellent, valid, special, and at the same time very human and close to patients, it was a great experience’.

On the other hand, few comments note that patients were not involved in clinical decisions and did not receive the expected patient-doctor communication on their health problem. The content of comments on hospital stay is different at 6 months and 1 year after hospital discharge. Specifically, patients’ focus moves towards follow up appointments for check-ups, comments on health problems, including fatigue and breathlessness or on physical improvements. Furthermore, at 6 months, respondents start discussing about drug adherence, few sentences as example:

‘I am regularly taking 1.25 mg bisoprolol in the morning and 5 mg in the evening’.

‘Since 2009 I take Cardioaspirin 100 mg and an integrator so that my cholesterol level is constantly under control’.

One year after the discharge patients’ comments shift towards self-care adherence in a broader way. Specifically, patients declare when they correctly adhere to self-care guidelines their healthcare status significantly improve:

‘If I pay attention to my body weight I’m better’.

‘I don’t take specific medication except aerobic exercise. I often do this kind of activity and it provides me a lot of benefit’.

A significant number of comments concerned people enhancement, specifically those who took care of patients both during the hospitalization and after the discharge. Immediately after the discharge, patients’ comments mainly focused on hospital professionals’ enhancement, expressing their kindness, professionalism, and humanity. Respondents’ enhancement shift on family and family members 6 months after discharge. However, some comments still expressed gratitude to professionals that took care of them during the hospitalization and still have the patient in charge outside the hospital. Family enhancement further increased in patients’ comments 1 year after the discharge.

In line, 6 months after discharge, respondents declared to be largely supported by family members in pharmacological and self-care behavior recommendations adherence. Comments revealed family members help patients in body weight control and in taking medicines:

‘My wife supports me in taking my drug therapy (including the administration of insulin), in maintaining relationships with the treating doctor, in the purchasing of drugs and in the regular intake at the scheduled times). My daughter follows specialist examinations by planning check-ups’

‘My wife helps me to control the weight and adjust the medicines’

The wife is often cited by men as preferred caregiver to support patients in pharmaceutical and self-care adherence, along with children. It is particularly clear 1 year after hospital discharge:

‘My wife gives me indispensable support in my daily routine’

‘I rely on my wife and daughter for symptom management. Drug therapies are regularly followed by my wife’

‘My daughter thinks about grocery shopping, medication, she books check-ups, and a woman comes to visit my house for the main cleaning a couple of times a week’

As for women responders, children or housekeepers are signalled as preferred caregivers when not able to carry out activities alone. Only a women reported to receive support from the husband:

‘I live with one of my daughters, I have another daughter, she helps me in cleaning and making food’

‘A housekeeper helps me every day, morning, and afternoon. She does the housework, she accompanies me to the medical exams and if I feel particularly tired, she takes me shopping’

The table below (Table 6) shows the most frequently observed words contained in the answers to the narrative questions at three different moments.

**Table 6:** Most frequently observed words contained in the answers to the narrative questions at three different moments

**Word Frequency**	**One month after discharge (T1)**	**Six months after discharge (T2)**	**One year after discharge (T3)**
What has positively or negatively impressed you during your hospital stay?	Professionals (*n* = 44) Availability (*n* = 28) Expertise (*n* = 20) Kindness (*n* = 17) Humanity (*n* = 7)	Check-ups (*n* = 7) Health problems, including fatigue, breathlessness (*n* = 7) Covid (*n* = 2) Health improvement (*n* = 2)	Health problems, including fatigue, breathlessness (*n* = 12) Health improvement (*n* = 2)
Please help us to value those people who have taken care of you: would you like to point out someone who has positively impressed you for the manners of taking care of patients?	Professionals (*n* = 15) Kindness (*n* = 11) Doctors (*n* = 9) Availability (*n* = 6) Professionalism (*n* = 6) Humanity (*n* = 4)	Family, including wife son and daughters (*n* = 6) Professionals (*n* = 5) Doctors (*n* = 4) Kindness (*n* = 4)	Family, including wife son and daughters (*n* = 10) Doctors (*n* = 7) Professionals (*n* = 5)
Would you like to tell us something about the support you receive from the people taking care of you?	–	Support/help/care for (*n* = 11) Family, including wife son and daughters (*n* = 9) Drug therapy (*n* = 5) Check-ups (*n* = 3)	Support/help/care for (*n* = 20) Family, including wife son and daughters (*n* = 15) Home care, including diet and Housekeeping (*n* = 12) Check-ups (*n* = 10) Drug therapy (*n* = 3)

## Discussion

Examples of chronic care pathway monitoring systems emerged in recent years, since assessing performance information at a pathway level emerged as an urgent need for worldwide healthcare systems [23, 35]. Indeed, chronic conditions are long-lasting in their effects, increasingly widespread, and exercise significant economic pressure on healthcare systems [36]. Among others, CHF is widely recognized as a chronic, progressive condition associated with significant morbidity, mortality, and health care expenditures [37]. Despite the features of this diseases, the above mentioned monitoring systems [23] lack in following the patients across the entire pathway, consequently grasping only some aspects although important. As an example, few moments and dimensions of the pharmacological adherence are measured, thus leaving a lack of awareness on the effective patient’s adherence across the whole pathway. Furthermore, those systems do not grasp the value generated for patients and communities, and by patients and communities as well. PROMs thus represent an RWE source embedded with the characteristic of continuously monitoring patients’ drug adherence in the daily life and across their pathway [18]. Both quantitative and qualitative results of the reported case study showed FTGM patients declare to strongly adhere to the CHF pharmacological therapy across the entire pathway [38], by confirming the excellent performance registered for statins as visible on the Interregional Performance Evaluation System (IRPES) [24, 38, 39] [40]. [41] This impressive result can be explained considering that, in this tertiary referral center, physicians and nurses dedicate a significant amount of time to properly inform patients. Furthermore, drug adherence results are in line with previous PROMs studies that reported high self-care in medication taking. However, as emerge from the literature, when analyzing PROMs results the bias of subjectivity should always be considered [42]. Although subjectivity may affect the results so as not to fully represent the current practice, the available research has shown that self-reported adherence can be regarded as robust data [43].

FTGM PROMs were also designed to measure an important but generally less careful adherence dimension, the adherence to self-care behavior recommendations. Indeed, adherence, both to drug therapies and self-care behavior recommendations, represent a key element in managing CHF and a key determinant of good health outcomes over time [40]. However, unlike medications adherence, the FTGM sample patients seemed less careful to adhere to these recommendations (e.g., physical activity). It thus worth focusing on Self-Care Maintenance results to better understand its different aspects and those elements that could help improving it, by maintaining the focus on the importance of all its adherence dimensions (i.e., individuals’ daily adherence to medication use, exercise, diet, and symptom monitoring). In the first follow up questionnaire, Self-Care Maintenance emerged as weakly correlated to Self-Care confidence. Such correlation seemed to become even stronger 7 months after the hospitalization. As reported by Riegel and colleagues [41, 44], Self-Care confidence mediates and/or moderates the Self-Care process. Similar results were found by Koirala et al. [45] when reporting that each 1-point increase in Self-Care confidence was associated with an increase in Self-Care Maintenance score by 0.3 (*p* = 0.000). The two dimensions can therefore not be seasoned as scissors when aiming at designing and implementing interventions to improve one and/or the other. Further noteworthy results emerged 7 months after the hospitalization. It clearly emerged that CHF patients that registered an outcome improvement were those who, on average, have had an improvement in the Maintenance. Conversely, those who experienced an outcome deterioration recorded a slight Maintenance improvement at 7 months and then stood at lower levels. Such an unexpected maintenance improvement step at 7 months could probably be explained by the fact that a follow up appointment was scheduled at 6 months, thus the patient received professionals’ counseling on the importance of treatment and recommendations adherence. As expected and according to the literature, CHF patients performing adequate Self-Care Maintenance are more likely to register outcome improvements [46–48]. Furthermore, those who increased confidence in adhering to therapies registered outcomes improvements. The Self-Care Confidence results as important as the Maintenance for outcome improvements. However, the Self-Care Maintenance and the Self-Care Confidence scores seemed to register different general trends over time. While the Confidence kept growing across the pathway - probably the continuous raise is associated with the patients’ CHF awareness over time, the Maintenance registered an initial growth and a registered a collapse 12 months after the hospitalization. Such drop could be explained by the possible lack of adherence counseling by professionals, considering that physicians exert substantial influence on patient adherence [49]. FTGM hospital specialists - the cardiologist working into the hospital - followed a slightly lower percentage of patients 1 year after the hospitalization (46.20%). The GP, who should oversee the patient when returning home, still followed a slight percentage of patients (30.4%). Open comments confirmed that family member took charge of all the patients’ adherence aspects from 6 to 12 months after the hospitalization. Respondents declared to be largely supported by family members in pharmacological and self-care behavior recommendations adherence. Comments revealed family members help patients in body weight control and in taking drug therapy. It is likely that, without the counseling of health professionals, the patients’ adherence may be reduced, or perceived as reduced. This result is twofold important. On the one hand, on the territorial assistance that deserves special attention for CHF: the considerable support provided to patients by family members seems to indicate a wide need of territorial assistance for CHF patients. Despite the clear need, only a small percentage of patients declared to have used home assistance services across the whole pathway. It therefore seems that many of the results achieved depend on the care and skill of hospital staff, despite the literature suggests that home care can provide a significant contribution to CHF patients’ outcomes in the long run [50]. On the other hand, the role of family members, caregivers, and the community emerged as key in producing value to CHF patients [51]. As reported in Pennucci and colleagues [52], the contribution of the single patient, caregiver or healthcare services’ user is determinant for reaching individual health outcomes, as well as to contribute to the sustainability of the system [52].

## Conclusions

The originality of the study consists in the chance of adopting a RWE source, specifically PROMs, as a longitudinal assessment tool that aims at supporting the monitoring and assessment of chronic care pathways to improve clinical practice.

The features of this PROMs collection and management model are relevant for researchers and policymakers as well as for front line staff and managers. Indeed, data collected via PROMs enable the healthcare system to adopt CHF patients’ personalized intervention along the pathway as part of the so-called personalized medicine. Since individual have unique characteristics at different levels (i.e., molecular, physiological, environmental exposure, and behavioral levels), they may need to have interventions provided to them for diseases they possess that are tailored to these unique characteristics. Personalized medicine implementation has been proven with the potential to increase patients’ quality of life and life extension [51, 53]. Furthermore, the continuous collection of open comments may activate co-production mechanisms, intended as an approach to ensure active participation of patients in designing, delivering, and evaluating public services. Indeed, co-production experiences are largely recognised as fundamental for improving healthcare services delivery [52, 54]. Despite some scholars highlighted the resistance of professionals and patients towards the patient involvement in the provision of care, it remains a key public strategy for achieving individual health outcomes, and for contributing to the sustainability of the healthcare system as well [55]. Managing collaborative process is as important as it is delicate [56]. Such strategies should not be as granted, and nevertheless they should be recognized as essential part of patient-centered policies [57]. For this reason, public policies might include mechanisms to stimulate and support healthcare professionals, patients and caregivers in their role of co-producers [58], in an individual level co-production process. This implies working (i) with healthcare professionals, in order to increase the awareness of the link between patient care and co-production, and their skills in educating both patients and caregivers to the entire spectrum of self-care, not only on adherence to therapies and maintenance, and (ii) with patients and caregivers, to make them more aware and self-confident of their role of co-producers and increase their individual knowledge, skills, and habits.

The continuous and longitudinal adherence monitoring enable the collection of key information for the implementation of intervention aiming at improving such pathway dimension. FTGM PROMs revealed limited patients’ adherence to self-care behavior recommendations (e.g., physical activity), thus emphasizing the importance of implementing interventions to create patients’ awareness on the importance of adherence to recommendations to achieve better health outcomes. Behavioral economics tools could be adopted to increase physical activity among CHF patients. Indeed, behavioral economics interventions focused at increasing elderly physical activity levels have been already tested in Tuscany and the experiences were successful as described by Pennucci et al. [59]. Among the tools to foster self-care behavior recommendations, social prescribing and peer to peer interventions could be implemented. Social prescribing, defined as professionals’ prescription of non-medical interventions (i.e., physical activity), was observed as a way to improve people wellbeing, reduce workload for healthcare professionals and the demand for secondary care services [60]. peer-to-peer activities can enhance medication adherence as well as other healthful behaviors, such as exercise [61]. Regardless of the chosen tool, when designing intervention to increase Self-Care Maintenance it is important to focus on the Self-Care Confidence’ mediation/moderation effect. It is therefore recommended that interventions on Maintenance are paired with those on the Confidence. Moreover, attention should be placed to the target of the intervention since those who have the most need to adhere to the recommendations are often those less willing to do so. Segmentation is a very effective way for finding well-defined homogenous groups in larger populations to effectively design and target behavioural change interventions, more effectively measure outcomes, address specific cognitive and behavioural patterns, and change behaviours of specific homogenous groups [62].

Policy makers and professionals should further explore which strategies are best suited to provide territorial assistance and ensure the patient’s full adherence even when the patient has no longer close contact with the hospital and its professionals. Caregivers emerged as playing a fundamental role in supporting CHF patients’ adherence both to medicines and self-care behavior recommendations, thus generating the typical phenomena in chronic diseases of high caregiver burden [63]. Interventions could be designed for supporting caregivers with home assistance and to promote the under-utilized home assistance and GP services. Since CHF patients are mostly men and frequently report the greatest demand for family assistance, specific interventions could be implemented to support their wives (or relatives) through territorial services dedicated to foster both healthcare and non-healthcare interventions adherence.

This study presents limitations that should be addressed and regarded as a basis for future developments. First, the reported results represent a picture of a specific center of excellence, the same survey applied in a different context (e.g., large generalist hospital) could produce different outcomes. Moreover, there are few benchmarking opportunities, since the Italian literature on the topic is still scarce, although the obtained results often coincide with the international literature. To conclude, the results of this study are not yet comparable with those data collected via FTGM electronic health records, therefore data reflect the patient’ perception even though their robustness was proven. It must be stressed, however, that the center is working on the connection of the two data sources, opening the chance for future studies that could combine the two data sources. Furthermore, it would be of great interest to link PROMs datasets to other RWE sources, such as those datasets collected across tele-medicine experiences in Lombardy region [64–66]. Despite the limitations, the model enables a large, longitudinal dataset to be collected over time, thus ensuring greater use as a management tool and for the benchmarking purposes. In the longer run, it is desirable that such continuous RWE monitoring systems would be to extend to other national and international hospitals and increasingly used to monitor and improve chronic patients’ adherence.

## Supplementary Information

**Additional file 1: Table S1.** SCHFI Scale. **Table S2.** PREMs questions considered across the analysis with the ones on adherence dimension.

**Additional file 2.**

**Additional file 3.** Detailed results for SCHFI items over time. Percentages of the options “Never or rarely” and “Always or daily” are reported.

## Data Availability

The datasets analyzed are available from the corresponding author upon reasonable request.
