# Supplementary material for: A longitudinal assessment of chronic care pathways in real-life: self-care and outcomes of chronic heart failure patients in Tuscany
Source: BMC Health Serv Res. 2022 Sep 10;22:1146. doi: 10.1186/s12913-022-08522-0 (PMC9463807; doi:10.1186/s12913-022-08522-0)
Supplement: Supplementary file 1 — Additional file 1: Table S1. SCHFI Scale. Table S2. PREMs questions considered across the analysis with the ones on adherence dimension. [file 12913_2022_8522_MOESM1_ESM.docx]

**Additional file 1.**

**Table S1.** SCHFI Scale

| SCHFI Scale | Never or rarely | Sometimes | Frequently | Always or daily |
| --- | --- | --- | --- | --- |
| Self-Care Maintenance (section A) |  |  |  |  |
| Weigh yourself? | 1 | 2 | 3 | 4 |
| Check your ankles for swelling? | 1 | 2 | 3 | 4 |
| Try to avoid getting sick? | 1 | 2 | 3 | 4 |
| Do some physical activity? | 1 | 2 | 3 | 4 |
| See your doctor or nurse? | 1 | 2 | 3 | 4 |
| Eat a low salt diet | 1 | 2 | 3 | 4 |
| Exercise 30 minutes? | 1 | 2 | 3 | 4 |
| Forget to take one of your medicines? | 1 | 2 | 3 | 4 |
| Ask for low salt items while eating out or visiting others? | 1 | 2 | 3 | 4 |
| Use a system to help you remember to take your pills? | 1 | 2 | 3 | 4 |
| Self-Care Management (section B) |  |  |  |  |
| How quickly did you recognize it as a symptom of heart failure? | 1 | 2 | 3 | 4 |
| Reduce the salt in your diet | 1 | 2 | 3 | 4 |
| Reduce your fluid intake | 1 | 2 | 3 | 4 |
| Take an extra water pill | 1 | 2 | 3 | 4 |
| Call your doctor or nurse for guidance | 1 | 2 | 3 | 4 |
| How sure were you that the remedy helped or did not help? | 1 | 2 | 3 | 4 |
| Self-Care Confidence (section C) |  |  |  |  |
| Keep yourself free of heart failure symptoms? | 1 | 2 | 3 | 4 |
| Follow the treatment advice you have been given? | 1 | 2 | 3 | 4 |
| Evaluate the importance of your symptoms? | 1 | 2 | 3 | 4 |
| Recognize changes in your health if they occur? | 1 | 2 | 3 | 4 |
| Do something that will relieve your symptoms? | 1 | 2 | 3 | 4 |
| Evaluate how well a remedy works? | 1 | 2 | 3 | 4 |

In this paper we considered section A, Self-Care Maintenance and section C, Self-Care Confidence that are presented to all the patients.

**Table S2.** PREMs questions considered across the analysis with the ones on adherence dimension

|  | T0  Baseline | T1  30 days | T2  6 months | T3  12 months |
| --- | --- | --- | --- | --- |
| During the last 6 months, who have mainly followed your care pathway? (GP, cardiologist, another specialist, no one in particular) | X |  | X | X |
| During the last 6 months, has your GP been in contact with the cardiologist to take care of you? | X |  | X | X |
| When you were discharged, did you receive clear information on…  -what to monitor once at home (physical exercise, food, smoking)  -which drugs to take  -what to do in case of complications  -who to call if you were in need  (physician, emergency department)  -which follow-up pathway to attend once discharged (disease rehabilitation) |  | X |  |  |
| Was your GP aware of your hospital admission? (Yes or no) |  | X |  |  |
| If yes, did your GP come to visit you during the hospitalization? |  | X |  |  |
| Is this your first hospitalization for heart failure? (Yes or no) | X |  |  |  |
| How long was your hospitalization?  - From 1 to 3 days  - From 4 to 6 days  - More than 6 days |  | X |  |  |
| After the discharge, did you used home care services? (Yes or no) |  | X | X | X |
