# Supplementary material for: A longitudinal assessment of chronic care pathways in real-life: self-care and outcomes of chronic heart failure patients in Tuscany
Source: BMC Health Serv Res. 2022 Sep 10;22:1146. doi: 10.1186/s12913-022-08522-0 (PMC9463807; doi:10.1186/s12913-022-08522-0)
Supplement: Supplementary file 2 — Additional file 2. [file 12913_2022_8522_MOESM2_ESM.docx]

**Additional file 2.**

- Before this hospitalization, how many different medications (i.e., medicines in packages with different names) should you usually take every day?
  - From 2 to 4
  - From 5 to 7
  - From 8 to 9
  - 10 or more
  - Don’t know
- Do you ever forget to take the medication your doctors prescribed you for CHF?
  - Yes
  - No
- If yes, how often do you forget to take the medication your doctors prescribed you for CHF?
  - Once a month
  - Once every two weeks
  - Once a week
  - Several times a week
