# Supplementary material for: A longitudinal assessment of chronic care pathways in real-life: self-care and outcomes of chronic heart failure patients in Tuscany
Source: BMC Health Serv Res. 2022 Sep 10;22:1146. doi: 10.1186/s12913-022-08522-0 (PMC9463807; doi:10.1186/s12913-022-08522-0)
Supplement: Supplementary file 3 — Additional file 3. Detailed results for SCHFI items over time. Percentages of the options “Never or rarely” and “Always or daily” are reported. [file 12913_2022_8522_MOESM3_ESM.docx]

**Additional file 3.**

Detailed results for SCHFI items over time. Percentages of the options “Never or rarely” and “Always or daily” are reported.

| Self-Care Maintenance (section A) | T0  Baseline | | T1  30 days | | T2  6 months | | T3  12 months | |
| --- | --- | --- | --- | --- | --- | --- | --- | --- |
|  | Never or rarely  (%) | Always or daily  (%) | Never or rarely  (%) | Always or daily  (%) | Never or rarely  (%) | Always or daily  (%) | Never or rarely  (%) | Always or daily  (%) |
| Weigh yourself? | 30.46 | 18.97 | 16.22 | 35.81 | 13.82 | 28.46 | 15.52 | 30.17 |
| Check ankles for swelling? | 24.71 | 23.56 | 12.84 | 47.97 | 8.13 | 47.97 | 8.93 | 43.75 |
| Try to avoid getting sick? | 19.65 | 17.92 | 15.44 | 41.61 | 12.90 | 50 | 13.91 | 47.83 |
| Do some physical activity? | 22.75 | 20.96 | 24.32 | 25 | 21.14 | 26.02 | 23.01 | 33.63 |
| See your doctor or nurse? | 4.65 | 59.88 | 4.03 | 77.18 | 7.26 | 75 | 5.26 | 79.82 |
| Eat a low salt diet | 14.04 | 24.56 | 5.44 | 35.37 | 8.13 | 30.89 | 8.77 | 39.47 |
| Exercise 30 minutes? | 54.97 | 9.94 | 47.97 | 16.89 | 34.68 | 15.32 | 38.26 | 16.52 |
| Forget to take one of your medicines? | 2.35 | 91.18 | 2.70 | 94.59 | 3.25 | 93.5 | 1.85 | 95.37 |
| Ask for low salt items while eating out or visiting others? | 29.24 | 15.20 | 21.48 | 18.12 | 23.58 | 18.70 | 15.79 | 22.81 |
| Use a system to help you remember to take your pills? | 36.63 | 47.09 | 28.86 | 59.06 | 27.42 | 58.87 | 36.52 | 49.57 |
| Self-Care Confidence (section C) | **T0**  **Baseline** | | **T1**  **30 days** | | **T2**  **6 months** | | **T3**  **12 months** | |
|  | Never or rarely  (%) | Always or daily  (%) | Never or rarely  (%) | Always or daily  (%) | Never or rarely  (%) | Always or daily  (%) | Never or rarely  (%) | Always or daily  (%) |
| Keep yourself free of heart failure symptoms? | 46.51 | 8.72 | 46.58 | 14.38 | 41.80 | 18.85 | 28.57 | 25.89 |
| Follow the treatment advice you have been given? | 0.58 | 75.72 | 1.36 | 81.63 | 0 | 78.05 | 0.88 | 76.99 |
| Evaluate the importance of your symptoms? | 13.37 | 41.28 | 6.94 | 44.44 | 9.02 | 43.44 | 14.91 | 46.49 |
| Recognize changes in your health if they occur? | 1.16 | 50 | 1.38 | 48.28 | 2.42 | 54.84 | 2.63 | 57.89 |
| Do something that will relieve your symptoms? | 19.19 | 33.14 | 19.05 | 33.33 | 20.33 | 32.52 | 18.58 | 36.28 |
| Evaluate how well a remedy works? | 8.24 | 38.82 | 9.79 | 39.16 | 11.57 | 38.84 | 11.50 | 44.25 |
